# A Goal-Directed Bayesian Framework for Categorization

**DOI:** 10.3389/fpsyg.2017.00408

**Published:** 2017-03-22

**Authors:** Francesco Rigoli, Giovanni Pezzulo, Raymond Dolan, Karl Friston

**Affiliations:** ^1^The Wellcome Trust Centre for Neuroimaging, Institute of Neurology, University College LondonLondon, UK; ^2^Institute of Cognitive Sciences and Technologies – National Research CouncilRome, Italy; ^3^Max Planck UCL Centre for Computational Psychiatry and Ageing ResearchLondon, UK

**Keywords:** Bayesian inference, goal-directed behavior, categorization, model comparison, accuracy complexity

## Abstract

Categorization is a fundamental ability for efficient behavioral control. It allows organisms to remember the correct responses to categorical cues and not for every stimulus encountered (hence eluding computational cost or complexity), and to generalize appropriate responses to novel stimuli dependant on category assignment. Assuming the brain performs Bayesian inference, based on a generative model of the external world and future goals, we propose a computational model of categorization in which important properties emerge. These properties comprise the ability to infer latent causes of sensory experience, a hierarchical organization of latent causes, and an explicit inclusion of context and action representations. Crucially, these aspects derive from considering the environmental statistics that are relevant to achieve goals, and from the fundamental Bayesian principle that any generative model should be preferred over alternative models based on an accuracy-complexity trade-off. Our account is a step toward elucidating computational principles of categorization and its role within the Bayesian brain hypothesis.

## Introduction

Adaptive behavior requires mapping single exemplars of stimuli or cues to their classes. This categorization saves time and computational resources, since it is unnecessary to learn specific actions for every exemplar (Rosch, 1973; Rosch et al., 1976; Barsalou, 1983). Furthermore, when novel stimuli are encountered, these can be assimilated into higher order classes, without the need to learn from scratch the behavioral response to each novel stimulus (Rosch, 1973; Rosch et al., 1976). For instance, consider the burden of learning an appropriate action (say, approach, or escape) for each new exemplar of animal encountered, such as a new predator or prey. Instead, we are able to immediately associate new animals with an appropriate category and to perform appropriate actions, like escaping a predator or approaching a prey.

The normative principles underlying categorization are the focus of rational theories. Some theories assume that categories are given *a priori* and that the stimulus-category mapping is learnt in a supervised way by presenting a set of stimulus-category pairs (Rosch, 1975; Homa et al., 1981; Nosofsky, 1986; Estes, 1994; Smith and Minda, 1998; Lamberts, 2000). A shortcoming of this perspective is the lack of an obvious explanation as to why category learning is adaptive for behavior. In other words, why should the brain organize stimuli into behaviorally meaningful classes?

One way to address this issue is to postulate an explicit mapping between categories and appropriate actions. For instance, the brain could learn to go/stop when a traffic light is green/red, irrespective of the specific traffic light cueing behavior. Based on this assumption (as in multi-attribute decision theory; Ashby and Townsend, 1986; Ashby and Gott, 1988; Maddox and Ashby, 1993), category learning corresponds to the acquisition of an adaptive stimulus-response mapping. However, one problem is that the appropriate stimulus-response mapping might vary across contexts, so that the same stimuli require particular actions in one context and other actions in another context (Barsalou et al., 2003; Barsalou, 2008). For instance, it is appropriate to run when a lion is encountered in the wild, while it is more appropriate to admire the same animal in a zoo. In cases like these, the stimulus category is constant, but the appropriate (context sensitive) action changes. In other words, a context-insensitive mapping between categories and actions can be problematic when the context determines which actions should be selected. This suggests that a model should accommodate the importance of context and should explicitly distinguish categories from actions (Barsalou et al., 2003; Barsalou, 2008; Rigoli et al., 2016a,b,c).

Another limit of the approaches described above is that categories (or responses) are established *a priori*. In other words, these formulations assume a pre-specified set of categories, while it is reasonable to expect that – at least in ecological situations – categories themselves need to be learnt from experience. Some models address this issue by adopting a framework in which categories emerge from clustering exemplars into groups (Anderson, 1990; Griffiths et al., 2007). This is achieved following normative principles such as the minimization/maximization of differences among stimuli within/between groups, and taking into account whether a large or small number of groups should be favored. However, these sorts of approaches – based upon stimulus statistics – do not consider the crucial link between categories and actions. Indeed, categories emerge from stimulus similarities independent of whether these similarities are action-relevant. Also, as for all other theories presented so far, categories are treated as context-invariant.

In summary, contemporary normative theories of categorization present at least one of the following shortcomings: categories are not learnt but established *a priori*; the stimulus-category mapping is independent of behavioral relevance; once learnt, the category structure and the stimulus-category mapping are context insensitive; categories and actions are conflated in such a way that a category always produces the same action. Here, we propose a new model of categorization that attempts to resolve these problems. We adopt a goal-directed perspective in which biological organisms aim to occupy certain goal-states associated with self-preservation (Rosenblueth et al., 1943; Miller et al., 1960; Dickinson and Balleine, 1994; Pezzulo and Rigoli, 2011; Botvinick and Toussaint, 2012; Solway and Botvinick, 2012; Friston et al., 2013, 2015; Pezzulo et al., 2013). This perspective stresses the importance of connecting category learning with behavioral relevance; insofar as classification is conceived as a means to achieve goals rather than a goal *per se*. We propose that agents have to categorize stimuli based on sensory evidence in order to select an appropriate behavior. At the same time, categorization requires computations that have benefits in terms of goal achievement but also costs (e.g., metabolic, opportunity costs, etc.) that need to be balanced against the benefits (Acuna and Schrater, 2009; Dolan and Dayan, 2013; Pezzulo et al., 2013; Shenhav et al., 2013; FitzGerald et al., 2014).

The above principles are formalized within a Bayesian scheme, where it is assumed that the brain builds a generative model of sensory cues that are relevant to achieve the organism’s goals. Indeed, from an enactivist perspective, the only *raison d’être* for perceptual synthesis and categorization is to inform action, which means that perception is itself inherently goal-directed. The generative model underlying active categorization is learnt through experience according to Bayesian model selection, which necessarily selects models with an optimal accuracy-complexity trade-off. This follows because (Bayesian) model evidence can always be expressed as accuracy minus complexity. Our main goal is to show that, from these basic normative principles, a generative model with certain characteristics arises where categories (1) are learnt based on their relevance for behavior, (2) are context-dependent in such a way that in different situations the same stimulus can be associated with one category or another, (3) are distinct from actions in a way that the same category can be associated with a certain action in one context and with another action in another context, (4) are organized hierarchically. Below, we present the theoretical arguments in detail and discuss how they relate to other models of categorization and with empirical research.

## A Theory of Categorization

Our theory follows recent formulations of the brain as a Bayesian statistics engine (Dayan et al., 1995; Knill and Pouget, 2004; Friston, 2005, 2010, 2012; Chater et al., 2006; Clark, 2013). We assume that the brain implements a generative model of sensory observations that is deployed to infer the latent causes of these observations (Courville et al., 2006; Gershman and Niv, 2010; FitzGerald et al., 2014). Our focus is on the characteristics a generative model should have, taking into account the nature of the physical world, the information relevant to achieve goals, and the biological constraints of the brain. Below, we compare different ways of instantiating the generative model and follow a method to evaluate these implementations in terms of their accuracy and complexity, following the principles of Bayesian statistics (Hoeting et al., 1999; Bishop, 2006).

Generative models can always be represented with the formalism of Bayesian graphs, in which nodes indicate variables and arrows indicate conditional dependencies (Bishop, 2006). All generative models comprise a perceptual layer of variables associated with causes or features (F) generating observations. Each node projects to an observation layer (O) that represents sensory input. While the perceptual layer corresponds to an estimate of the (hidden or unobserved) features, the observation layer represents the sensory evidence for these features, with the two layers connected in a probabilistic fashion. In our formulation, a decision problem (i.e., choosing an appropriate action) is cast in terms of inferring the most likely action via a posterior distribution over allowable actions. This (planning as inference) approach has been described in detail previously (Pezzulo and Rigoli, 2011; Botvinick and Toussaint, 2012; Solway and Botvinick, 2012; Friston et al., 2013, 2015). Here, an action variable (A) and a goal state variable (G) are included in the generative model. The latter can assume two values corresponding to whether a goal state (or reward) has been attained or not. During inference, goals are treated as observations (generally, as the prior probability distribution or belief) and make it possible to infer the action that is most likely given the goals (or, more generally, the prior belief that goals will be attained or experienced). Note that action is not an observable outcome but a (hidden) cause of goals and other observations. This means action has to be inferred and can be thought of as a policy or plan.

**FIGURE 1:**
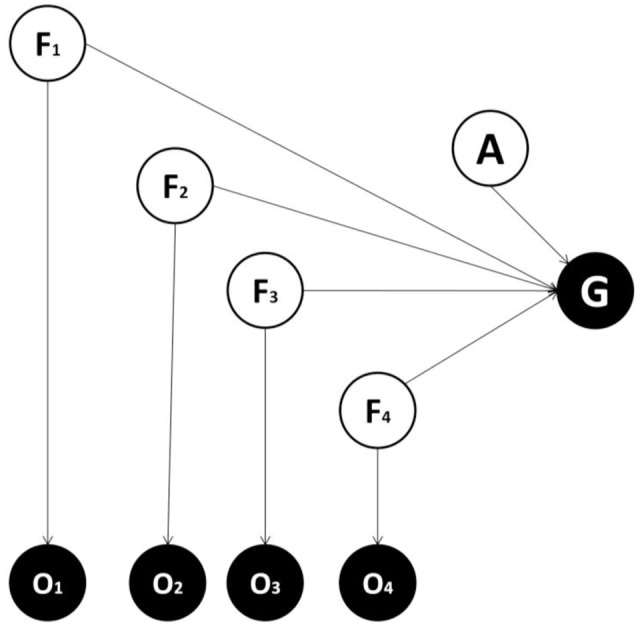
**Generative model used in the first alternative instantiation.** A generative model is essentially a probabilistic description of how causes generate consequences. Circles and arrows represent random variables and conditional dependencies respectively. White and black circles represent non-observed (causes) and observed variables (consequences) respectively. F = features variables (four are considered in the example), which represent hidden causes of feature of the world that generate outcomes observations; O = observation variables, one per feature, which provide sensory evidence about the features; A = action variable; G = goal variable. The goal variable is an observable outcome, conceived as equal to one when a goal is achieved and zero otherwise. In this implementation, feature variables map directly to the goal without any intervening latent variables.

**FIGURE 2:**
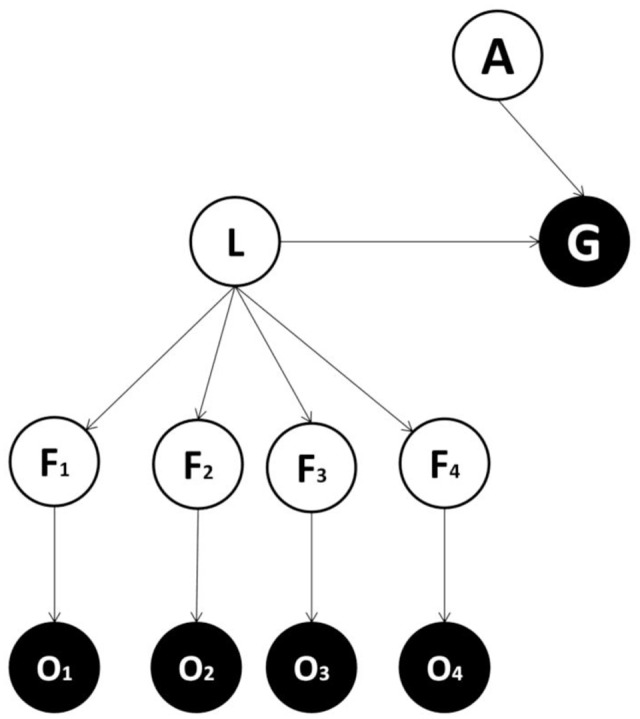
**The second form of generative model.** This form is the same as in the first implementation except that it includes: L = latent variable, which represent the latent cause of sensory features. The latent variable – but not the single features variables – project to (or cause) the goal state.

**FIGURE 3:**
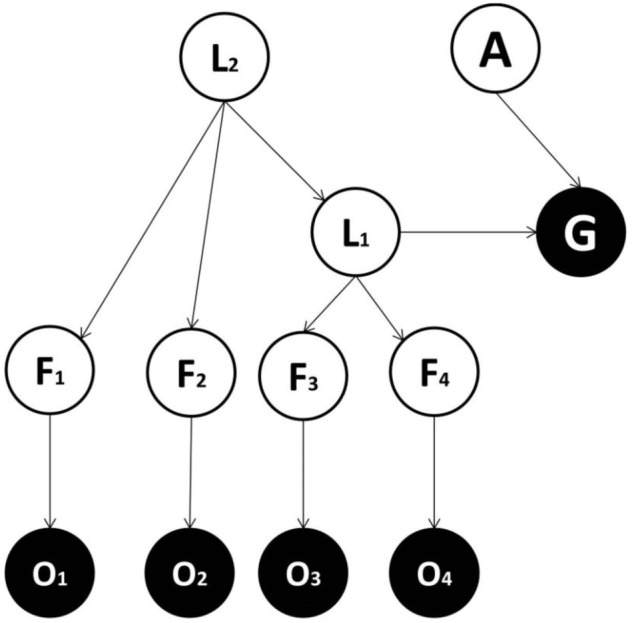
**Generative model used in the third implementation.** This is the same as the model used in the second implementation, except that it entails a hierarchical structure: L_1_ = lower-order latent variable, representing more specific classes; L_2_ = higher-order latent variable, representing more generic classes. L_2_ projects to a subset of features (e.g., F_1_ and F_2_) and to L_1_. L_1_ projects to other features (e.g., F_3_ and F_4_) and to the goal state.

**FIGURE 4:**
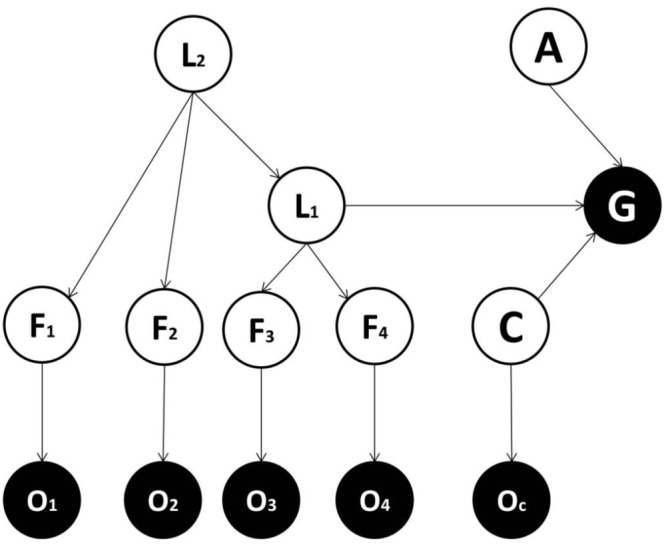
**Generative model used in the fourth implementation.** This is the same as the model used in the third implementation except that it comprises contextual effects: C = context variable; O_C_ = the sensory evidence about the context variable.

We consider alternative forms for generative models and compare them in terms of the accuracy/complexity trade-off, to establish which might be favored in ecological circumstances (Hoeting et al., 1999; Bishop, 2006). In one candidate form (**Figure 1**), there is a direct mapping from features to the goal state, while in other models (**Figures 2–4**) a categorization subsystem is inserted between goal states and features. This categorization layer includes variables representing the latent causes of features (where features cause observations – including observed goals). The categorization subsystem is either organized hierarchically (**Figures 3**, **4**) or not. In hierarchical forms, a lower-order (and more specific) latent variable (L1) project to a subset of feature variables and a higher-order (and more general) latent variable (L2) project both to lower-order latent variables and to other feature variables. Among models with a hierarchical structure, a context variable (C) can be considered explicitly (**Figure 4**) or not (**Figure 3**) and encodes different mappings from latent variables (and action) to the goal variable.

Below, we compare and contrast these different forms and evaluate them according to Bayesian statistics; namely, in terms of their accuracy and complexity (Hoeting et al., 1999; Bishop, 2006). The former aspect relates to the ability of the generative model to infer reliably the appropriate action, while the second component relates to the number of parameters used to explain observations. In keeping with Bayesian principles, we will argue that an implementation with a similar level of accuracy but a smaller degree of complexity (i.e., smaller number of parameters) should be preferred. As noted above, maintaining a high accuracy while minimizing complexity costs produces a model with greater evidence (and minimizes its variational free energy), in accord with Ockham’s principle (and the free energy principle).

Crucially, our formal implementations present different degrees of factorization, which changes the number of variables and implicit complexity. In other words, introducing latent variables can result in a smaller number of conditional dependencies and therefore in a smaller number of (effective) model parameters. Whether an implementation with high degree of factorization should be favored depends on its accuracy; i.e., on whether it provides a parsimonious but accurate explanation for outcomes (including goals). The key argument here is that the state-action-outcome contingencies which unfold in the real world mean that generative model with the greatest evidence (i.e., selected by evolution and learning) must be characterized by a certain factorization structure – which should be reflected in the brain structure (and function).

Before a particular generative model can be used for inference, its structure (i.e., the variables, states, and conditional dependencies) needs to be learnt. We will not go into the processes underlying this (structure) learning. Here, our focus is on the outcome of learning; namely, on justifying formally why a certain model structure should, eventually, emerge from the statistical structure of the world.

### Why is Categorization Important?

Our perspective casts the fundamental computational problem facing agents as inference in relation to an appropriate action, given an expectation of goal attainment and evidence for sensory features (Pezzulo and Rigoli, 2011; Botvinick and Toussaint, 2012; Solway and Botvinick, 2012; Friston et al., 2013, 2015). One simple way to approach this problem is to use the generative model shown in **Figure 1** (Model one). This includes several feature variables in the perceptual layer, each projecting to one observation node, plus an action variable and an observable goal-related outcome. Crucially, in this generative model, feature variables map directly to the goal state. This differs from the second generative model (Model two; shown in **Figure 2**), where feature variables are caused by a latent variable, which in turn maps to the goal state (see also Courville et al., 2006; Gershman and Niv, 2010; FitzGerald et al., 2014). Which of these two models is more plausible? The answer to this question depends on the true causal structure of the environment (Collins and Frank, 2013; Stoianov et al., 2015). If features are independent of each other and different combinations of features need to be considered to select an appropriate action, then adding a latent variable does not help as either (i) the action selection process would be less accurate or (ii) the latent variable would require at least as many states as combinations over features to achieve a sufficient level of accuracy, thus inducing a larger number of model parameters (and hence more complexity). Consider instead an alternative scenario, where the true features depend on the same latent variable. In the case the state of the latent variable, rather than the state of single features, is sufficient for inferring an appropriate action, a model with latent variables is able to obtain the same level of accuracy with minimal complexity, and will be selected on the basis of its evidence. Note that this is ubiquitous in ecological situations. For instance, a prey only needs to infer the presence of a lion rather than the lion’s individual characteristics; such as golden fur, long teeth or a brown crest.

When true contingencies follow a latent cause structure, a model that recapitulates this structure should be favored (by natural selection, over evolutionary time and by the brain, over somatic time) because of the smaller complexity cost. One can show this formally by focusing on parameters of the models that distinguish one model from another. We will refer to these as *characteristic parameters*. In what follows, we will associate the complexity with the number of parameters. Strictly speaking, complexity is the Kullback–Leibler divergence between the posterior and prior distributions over model parameters; however, in the context of a large number of observations, complexity can be shown to increase with the number of free parameters. Indeed, this is the approximation used by information criteria, such as the Akaike and Bayesian information criteria (used in model selection). Furthermore, to simplify things we will only consider the parameters encoding beliefs about variables. Technically, these can be regarded as the parameters of multinomial distributions over different sets of categorical variables.

Under these simplifications, the characteristic parameters of models one and two comprise all their parameters except those related to the feature-observation mapping. Model one has P_1_ = $n_{f}^{F}$n_a_n_g_ characteristic parameters, while model two has P_2_ = $n_{f}^{F}$_L1_ + n_L1_n_a_n_g_, where F is the number of features, n_f_ is the number of states for each feature (for simplicity, here we assume equal number of states for all features), n_a_ is the number of states for the action, n_g_ is the number of states for the goal variable, and n_L1_ is the number of states for the latent variable L1. If the condition P_2_ < P_1_ applies, then with simple algebraic transformations we obtain:

$$\begin{array}{c} n_{L1}<\frac{n_{f}^{F}n_{a}n_{g}}{n_{f}^{F}+n_{a}n_{g}} \end{array}$$

Taking the minimum values allowed for the quantities on the right-hand side – namely, *F* = 2, n_f_ = 2, n_a_ = 2 and, n_g_ = 2 (because the minimum number of levels for a categorical variable is 2) – the term on the right side of the formula is equivalent to two, and hence this is necessarily equal to or smaller than n_L1_; because L1 cannot have less than two states. Therefore, in this case, Model one has the same or a smaller number of parameters than Model two, and there is no reason to justify Model two. However, if just one of the values on the right side of the formula is larger than two, then n_L1_ can be smaller than the term on the right side, and Model two will be favored; provided that it reflects true environmental contingencies.

In sum, a generative model, in which several features are mapped to a latent cause, is preferable in situations where this model reflects true contingencies (Pezzulo and Calvi, 2011). It is important to note that this condition is likely to be the rule rather than the exception for biological systems. This is because features are attributes of objects. For instance, color, shape, and movement characteristic of a certain predator are usually experienced together because they are caused by the presence of the predator (a latent variable). This justifies the notion that entertaining a generative model that contains latent variables reduces complexity and is an ecological optimal strategy – insofar as it allows organisms to suppress computational costs, while maintaining the same accuracy. This, we argue, is the basic normative principle behind categorization.

### Why Are (Deep) Hierarchical Models Important?

We now consider whether, and under which conditions, a generative model with a hierarchical structure is favored. We compare Model two (see **Figure 2**), with no hierarchy and Model three (see **Figure 3**), where latent cause variables are organized hierarchically. The latter comprises a higher order (and more general) latent variable (L2) which causes a lower order (and more specific) latent variable (L1), in turn connected to the goal variable. The question addressed is whether a hierarchical structure is useful and under which conditions. Consider having to categorize an animal based on a noisy observation of five features (head, body, teeth, nails, and limbs), each having two possible states (small and big). Imagine that just by seeing the shape of the limbs, teeth and nails, it is possible to distinguish between herbivorous and carnivorous animals. At the same time, other information (i.e., the shape of head and body) cannot distinguish between herbivorous and carnivorous animals. This represents an example where a hierarchy is more parsimonious because (i) a subgroup of features is sufficient to classify stimuli into (action-relevant) general classes (herbivorous and carnivorous), (ii) another subgroup of features (the shape of head and body) is irrelevant for the general classification, but maybe useful for a more specific classification (the animal identity). As above, these considerations only apply if they are reflected in the real contingencies generating observations.

To demonstrate the advantages of hierarchical organizations, we focus on the characteristic parameters of the models, which in this case comprise all parameters except those characterizing the link among the higher order latent cause variable L1, the action, and the goal, since these are common to both models. Model two has P_2_ = $n_{\mathrm{f1}}^{F1}$$n_{\mathrm{f2}}^{F2}$_L1_ characteristic parameters, where F1 and F2 are the number of features associated with L1 and L2 respectively, and n_f1_ and n_f2_ are the number of states for each feature for the first (linked with L1) and second (linked with L2) subset of features respectively (for simplicity, we assume equal numbers of states for all features in each subset). Model three has P_3_ = $n_{\mathrm{f2}}^{F2}$_L1_n_L2_ + $n_{\mathrm{f1}}^{F1}$_L1_ characteristic parameters, where n_L2_ is the number of states for the higher order latent cause L2. If the condition P_3_ < P_2_ applies, then we obtain:

$$\begin{array}{c} n_{L2}<\frac{n_{f1}^{F1}n_{L1}(n_{f2}^{F2}-1)}{n_{f2}^{F2}n_{L1}} \end{array}$$

Taking the minimum values allowed for the quantities in the right-hand side: F1 = 2, F2 = 2, n_f1_ = 2, n_f2_ = 2, n_L1_ = 2, the term on the right side of the formula is equivalent to one, and hence this is necessarily smaller than n_L2_; because L2 cannot have less than two states. Therefore, in this case, Model two has necessarily a smaller number of parameters than Model three, and there is no reason to justify Model three. However, for instance considering the same values for the quantities on the right side of the formula except that now F2 = 3, the right side of the formula is equal to 3.5 and allows n_L2_ to be smaller than the term on the right side, so that Model three can be favored if it reflects the true environmental contingencies. For instance, if we go back to the example above requiring the categorization of animals based on visual cues, it is straightforward to show that Model two would need 64 characteristic parameters (assuming two possible animal identities, i.e., two states for L1), while Model three would only need 40 characteristic parameters.

In sum, a hierarchical categorization structure should be selected when certain, and not other, features are linked with more general classes and need to be integrated with a second group of features for more detailed perceptual classification. Note that this condition characterizes most ecological situations.

### Why Are Context and Action Important?

Recall the previous example, where categorization was based on a noisy observation of four features (head, body, teeth, and nails), each having two different possible states (small and big). However, now we focus on the explicit action called for by each category, for instance deciding whether to approach or avoid the animal. Furthermore, the correct action depends on the context, for instance avoiding a lion is appropriate in the wild and approaching a lion is appropriate at the zoo. In this situation, a model that does not include a context variable (Model three) has to consider the context by adding further states to the latent variable L1. In other words, in order to model the influence of being in the wild or at the zoo, the number of possible states of L1 would correspond to the number of possible animals times two; i.e., the number of possible contexts (wild and zoo). This increases complexity dramatically, favoring a model where context is modeled as in Model four. Intuitively, if two variables (e.g., context and content) can be factorized one can either encode their joint occurrence with (marginal) probabilities over both, or encode the joint distribution with a larger number of parameters. Clearly, the former is much simpler and will afford the same inference; provided the two variables are statistically independent. A nice example of this is the segregation of dorsal and ventral pathways in the brain to encode *what* and *where* features (Friston and Buzsáki, 2016; Mirza et al., 2016). The key thing here is that knowing *what* an object is does not depend (on average) on *where* it is in the visual field – and *vice versa*.

To prove this more formally, we focus on the characteristic parameters of Model three and four, which comprise all parameters except those pertaining to the feature-observation mapping and the mapping from L2 to the second subset of features (since these are common to both models). Model three has P_3_ = $n_{\mathrm{f1}}^{F1}$_L1_n_g_n_a_ characteristic parameters. However, a contextual variable needs to be considered to achieve a sufficient level of accuracy, and in Model three this influences n_L1_, which now corresponds to n_L1_ = n_l1_n_c_, where n_L1_ is the number of original states in the latent variable and n_c_ is the number of states for the context variable. Hence, we can rewrite P_3_ = $n_{\mathrm{f1}}^{F1}$_l1_n_c_n_g_n_a_. Model four has P_4_ = $n_{\mathrm{f1}}^{F1}$_l1_ + n_l1_n_c_n_g_n_a_ characteristic parameters. Taking the minimum values allowed for the variables above: F1 = 2, n_f1_ = 2, n_l1_ = 2, n_c_ = 2, n_g_ = 2, n_a_ = 2, then P_3_ = 64 and P_4_ = 24, therefore Model four can always have less parameters than Model three, and should be selected when it models the true environmental contingencies.

A similar point can be made to justify the inclusion of the action variable. So far we have not discussed the role of the action variable explicitly. We can reason that, with a one-to-one mapping from latent variables to actions, in other words if the same action is always correct given a particular latent cause, there is no real need to add another variable to account for action, as this would just increase complexity. However, with the introduction of a context variable, which establishes whether one action (e.g., approach or escape) should be performed or not for any given stimulus, introducing an explicit action variable reduces complexity. Indeed, precluding action as an explicit variable would entail merging the action variable with the latent variable L1, again increasing complexity compared to a model where action is modeled explicitly. As above, this argument rests upon the statistical independence or factorization of the probability distributions over action and latent variables (conditional upon any context).

More generally, the inclusion of an action and goal state variable highlights the purposeful and active nature of categorization. This is most evident in the fact that, in our scheme, inference is necessary to estimate the posterior probability over actions, and therefore a generative model is only useful when an action needs to be selected; a condition requiring that (1) more than one action is available and (2) only sensory features relevant for behavior are included in the generative models. The first point implies that one action can be optimal in one condition and another action in another condition. The second point implies that sensory features that are never action-relevant can be disregarded, explaining for instance why the perceptual system of biological organisms ignores a large portion of the visual and auditory spectrum.

In summary, when two variables – one assigned the role of latent variable and the other the role of context – are statistically independent and when the correct action depends on their joint occurrence, the brain should select a generative model that represents both the latent and context variable; thereby enabling efficient inference about action. Finally, the inclusion of an explicit action variable within the generative model is important to highlight the active nature of inference and categorization.

### Model Predictions

Our model has clear implications for research in the laboratory – and here we present some key empirical predictions. First, our model accounts for the evidence that living creatures can learn categories from repeated experience with exemplars. This is not trivial especially as some influential models of categorization (Rosch, 1975; Homa et al., 1981; Nosofsky, 1986; Estes, 1994; Smith and Minda, 1998; Lamberts, 2000) apply only to situations where categories are given *a priori* (though note other models account for category learning). A second prediction of our framework is that category learning will be highly nuanced by action. For example, consider two sets of stimuli with similar perceptual features. In a condition where both sets require performance of the same action, our model predicts that the brain will represent both sets within the same category. On the contrary, in a condition where sets of stimuli call for different actions, our model predicts that the brain will represent these sets as distinct categories. Though empirical evidence on this and similar scenarios is scarce (Roach et al., 2017), investigating this prediction seems relatively straightforward as in, for example, asking participants to categorize a stimulus presented following another stimulus. Our prediction is that prior exposure to a stimulus will facilitate categorization to a higher degree if the two stimuli have been previously associated with the same action. Notably, this effect is not predicted by models of categorization based on the notion that categories are learnt exclusively from perceptual features (Anderson, 1990; Griffiths et al., 2007).

Along these lines, our model proposes also that categories will be formed based on associated goals, beyond perceptual similarity and stimulus-action associations. This is analogous to the notion of *ad hoc* categories (Barsalou, 1983), which are built around common goals (e.g., “things to sell at a garage sale” or “things to take on a camping trip”) and not around perceptual similarities. Research has supported the relevance of this type of categorization (Barsalou, 1983; Barsalou, 2008), providing evidence which is hard to reconcile with theories based on perceptual features only (Anderson, 1990; Griffiths et al., 2007).

Another prediction derived from our model relates to the fact that categories tend to be organized along a hierarchy, a prediction consistent with empirical observations (Collins and Quillian, 1969; Warrington, 1975). A hierarchical organization is not easy to reconcile with many influential models of categorization (Rosch, 1975; Homa et al., 1981; Ashby and Townsend, 1986; Nosofsky, 1986; Ashby and Gott, 1988; Anderson, 1990; Maddox and Ashby, 1993; Estes, 1994; Smith and Minda, 1998; Lamberts, 2000). In our model this hierarchical aspect derives from the fact that some features are linked with more general classes and need to be integrated with a subordinate set of features for more detailed perceptual classification.

One final prediction concerns the role of context. Several influential models of categorization conceive categories as rigid entities that remain fixed across different contexts. On the contrary, our model predicts that categories will be highly context-dependent (Barsalou et al., 2003; Barsalou, 2008). This implies that the same stimulus can be associated with a set of objects in one context and with a different set of objects in another. Studies that directly elucidate the role of context during categorization are rare (Barsalou et al., 2003; Barsalou, 2008), but our emphasis on context-dependency is indirectly supported by research on long-term memory (Tulving and Thomson, 1971; Squire, 1986; Tulving, 2002), classical conditioning (Bouton, 1993), and evaluation processes (Rigoli et al., 2016a,b,c).

In summary, we argue that the modeling approach adopted in this paper can motivate empirical research in several directions, and can offer insights for interpreting available evidence. Here, we have provided some examples that speak to novel and specific empirical predictions arising from our theoretical proposal.

## Discussion

Categorization is a fundamental faculty of intelligent agents, enabling them to save computational resources (eschewing the storage of appropriate actions for all stimuli) and generalize an adaptive behavioral repertoire to novel stimuli (by assimilating new stimuli into extant categories) (Rosch, 1973; Rosch et al., 1976). We propose a normative account of categorization, based on fundamental Bayesian principles. Our premise is that the brain (and natural selection) builds a generative model of sensory inputs (that includes preferred outcomes or goals), which is acquired according to the rules of Bayesian model selection. This involves maximizing Bayesian model evidence (or minimizing variational free energy) and entails trading-off accuracy and complexity (Acuna and Schrater, 2009; FitzGerald et al., 2014). This provides an opportunity to minimize complexity given the nature of probabilistic encoding where the joint distribution over statistically independent variables can be factorized in terms of their marginal distributions. This factorization enables an efficient (minimally complex) generative model that entails latent variables arranged hierarchically together with explicit variables representing action and context.

Ultimately, these structural characteristics emerge from a very simple consideration; namely, that they reflect the state-action-outcome contingencies that unfold during exchanges with the environment. Indeed the environment is characterized by objects which correspond to sets of correlated features. This motivates the inclusion (in the generative model) of latent variables that generate certain feature patterns and represent objects (Courville et al., 2006; Gershman and Niv, 2010; FitzGerald et al., 2014). Objects share features with other objects – and this represents the basis for more general (hierarchical) classification possibilities. Here, we acknowledge the importance of taking these general classifications into account through a hierarchical scheme of latent causes, a further instance of factorization. In addition, in the real world, whether an action is appropriate given a certain object (i.e., the objects affordance) often depends on the presence of other environmental features, which can be conceived as context (Barsalou et al., 2003; Barsalou, 2008; Rigoli et al., 2016a,b,c). This speaks to the explicit representations of action and context in the generative model. Overall, we propose that the real nature of the world and interaction between the objects and the agent is the reason why the brain (and evolution) has selected generative models for categorization with the characteristics described here, under the assumption that the brain (and evolution) follows Bayesian principles.

According to the Bayesian brain hypothesis, the basic tools needed to perform inference (namely, the ability to generate, implement, select and use generative models) must be supported by the brain’s anatomy and physiology. The constant interchange between the brain, the body and the environment is what favors the selection of generative models – that necessarily show increasing consistency with the agent-environment interaction. Therefore, it is reasonable that, during development, generative models become more and more accurate (under the constraint of minimizing complexity). However, note that our proposal implies that categorization performance will be *sufficiently* good, but (as with models of bounded rationality) will not follow formal logic rules (Oaksford and Chater, 2007). This is expected for at least three reasons. First, the brain’s physiological constraints and bounded computational resources limit the ability to model every environmental contingency, entailing a systematic discrepancy between inferred and true environmental dynamics. Second, prior beliefs are often adaptive but may also induce biases and errors if environmental contingencies change abruptly. Indeed, the very process of learning is a testament to the persistent suboptimality of our generative models. Third, the brain is unlikely to perform exact Bayesian inference (or model selection), but is more likely to implement approximate Bayesian inference (and selection), such as free energy minimization (Friston, 2010), which is based on factorization of posterior beliefs (known technically as a mean field approximation). In short, *optimality* in approximate Bayesian inference is a nuanced optimality that probably accounts for the intractable nature of exact Bayesian inference.

One especially important constraint is the agent’s action repertoire, as ultimately categories are ways to classify objects in behaviorally relevant ways. This is captured by our scheme, in which sensory and action components are integrated within a unifying generative model and action selection drives – and is driven by – inference. The latter aspect explains the selective nature of categorization insofar as sensory features irrelevant for action selection are disregarded (i.e., are not a necessary component of the generative model). Furthermore, this implies that perceptual categorization should not be seen in conditions where a single action is available.

Our account is closely related to previous normative models. The proposal that categorization has a functional role – in saving computational resources and generalizing adaptive behavior to novel stimuli – can be already found in the seminal work of Rosch (1973, 1975) and Rosch et al. (1976). Early computational theories focus on the rules guiding the attribution of novel stimuli to appropriate categories (Rosch, 1975; Homa et al., 1981; Nosofsky, 1986; Estes, 1994; Smith and Minda, 1998; Lamberts, 2000). We build on these theories by proposing a Bayesian approach, which allows us to derive fundamental proprieties of categorization, such as its link with action and context and its hierarchical nature. A link with behavior has been previously highlighted in multi-attribute decision theory (Ashby and Townsend, 1986; Ashby and Gott, 1988; Maddox and Ashby, 1993) that casts categorization as partitioning a multidimensional space of attributes based on the correct response associated with each partition. This can be conceived as a more general framework compared to the one pursued here; because it makes minimal assumptions about the stimulus-action mapping. While this can apply to any categorization problem, we restrict our focus to real-world contexts. Our proposal is that the statistical proprieties linking action with the world encourage the brain to develop or select generative models with the characteristics described here; namely, constraining the possible stimulus-action mappings relevant for biological systems.

Recently, machine learning has seen an impressive development in classification algorithms based on Bayesian statistics. This approach has dramatically improved solving problems relevant for categorization, like object recognition. Interestingly, some of these algorithms use methods such as neural networks which call on aspects of neurobiology and highlight a similarity with brain functioning (e.g., Bishop, 2006; Hinton et al., 2006; Anthony and Bartlett, 2009). However, one limit is that the interpretation of the underlying mechanisms is sometimes difficult. Indeed, despite excellent performance, it is not straightforward to isolate the processes underlying the functioning of many neural network algorithms (for instance, how information is represented and processed), which is why these models are often described as “black boxes” (Sjöberg et al., 1995). Therefore, despite the importance of machine learning, we believe that to investigate cognitive and biological systems, it is equally important to study models where the underlying processes can be isolated and understood in terms of explicit generative models and Bayesian (variational) principles.

Our view is formally connected with recent proposals that formulate decision-making and motor planning as Bayesian inference (Pezzulo and Rigoli, 2011; Botvinick and Toussaint, 2012; Solway and Botvinick, 2012; Friston et al., 2013, 2015; Rigoli et al., 2016b). In this perspective, action selection corresponds to inferring the posterior probability of an action based on a generative model, given evidence about the current state, and based on “fictive” evidence about the future states and, in some formulations, based on prior beliefs about future outcomes (Friston et al., 2013, 2015). These prior beliefs or preferences can be interpreted as the organism’s goals (cf. our goal variable above). An in-depth discussion of this approach, compared to standard decision-theory formulations, goes beyond the scope of the manuscript. However, the reason for using this perspective here relies on the possibility of leveraging on the principles of Bayesian model selection (i.e., the accuracy-complexity trade-off) to explain why certain generative models better explain agent-world exchanges – and thus provide an explanation as to why particular model forms appear to be employed by the brain. Though it might be possible to cast our reasoning in terms of standard decision-theory formalisms, using a Bayesian formulation appears a direct way to unify the inference and decision steps and exploit the accuracy-complexity trade-off principle to explain categorization, cortical hierarchies and, indeed, functional segregation in the brain (see below).

Our model fits particularly well with Active Inference (Friston et al., 2013, 2015; FitzGerald et al., 2014), a general formulation of brain function, proposing an integration of perception and action is explained by approximate Bayesian inference (or equivalently, variational free minimization). A strong link between Active Inference and the present theory is the integration of action and perception within a unifying Bayesian inference scheme. In addition – though our focus is not on the mechanisms underlying inference and learning – we stress that free-energy minimization is an important candidate for the inference processes mentioned here, and has several advantages in terms of computational efficiency and biological plausibility (Friston et al., 2013, 2015; FitzGerald et al., 2014).

Our theory is also closely related to some recent computational models of cognitive control (Collins and Frank, 2013; Stoianov et al., 2015). These implementations conceive task sets (i.e., the mapping from stimuli to action associated with reward) as latent causes that need to be inferred from the observation of stimuli (called context) with a Dirichlet process (Anderson, 1990; Griffiths et al., 2007). Common characteristics of this and our theory are the explicit role of action selection and the separation of stimuli from their latent causes. In addition, these models propose an explicit mechanism for learning (i.e., the Dirichlet process). Here we pursue a different argument; as we appeal to the principles of Bayesian model selection to justify certain characteristics of the generative model, with some characteristics common to the other theories (Collins and Frank, 2013; Stoianov et al., 2015) and other characteristics peculiar to our formulation (for instance, the inclusion of multiple perceptual features and the hierarchical organization of latent causes).

One aspect of our theory is the importance of latent causes. This has been stressed already in recent computational proposals (Courville et al., 2006; Gershman and Niv, 2010; Collins and Frank, 2013; FitzGerald et al., 2014; Stoianov et al., 2015; Rigoli et al., 2016b). Our contribution clarifies further why latent causes are important; namely, because they reduce the complexity of the generative model by simplifying the encoding of joint probabilities over features and action. For instance, it is redundant to consider the shape, color, and smell of an animal independently. It is better to first infer from these features the animal’s identity – e.g., whether it is a predator – and then select an appropriate action. The converse approach (namely, to directly infer the features that explain every detail of sensory data) is suboptimal. In statistics, this failure to minimize complexity is known as ‘overfitting’ and, invariably, leads to a failure of generalization, when the model is confronted with new data.

Another key characteristic of our proposal is the hierarchical organization of the generative model. The idea of a hierarchical category system has a long history, going back to Aristotle. In cognitive science, it was proposed by early propositional models of semantic representation (Collins and Quillian, 1969; Warrington, 1975); where a hierarchy enabled one to store a property shared by several objects only once (at the level of an higher-order class) hence saving computational resources. With the more recent criticism of propositional models, the interest in explicit hierarchical schemes has declined (McClelland and Rogers, 2003). For example, several prominent normative models of categorization do not consider any hierarchy (Rosch, 1975; Homa et al., 1981; Nosofsky, 1986; Estes, 1994; Smith and Minda, 1998; Lamberts, 2000). Here, we reprise this concept within a new framework, where latent causes are arranged hierarchically. The motivation for implementing a hierarchy is the suppression of complexity; because some features of the stimulus can be captured by a simple higher order category. Note that hierarchies can be potentially organized into multiple levels, accounting for the fact that classification can be represented along a taxonomy tree (e.g., from a living organism, an animal, a mammalian, a dog, to my dog). This aspect of our model resonates with the original idea of a categorical hierarchy and sheds new light on its characteristics. The same justification of hierarchical models, advocated here in relation to features, can be generalized to action and goal variables, establishing a link, for instance, with hierarchical reinforcement learning (Barto and Mahadevan, 2003; Botvinick et al., 2009; Rigoli et al., 2016b). Our argument is also relevant for neurobiological theories of hierarchical brain organization (Friston, 2005, 2010; Pezzulo et al., 2015), raising the possibility that the functional segregation might reflect distinct representations of latent causes or clusters of latent causes, which are independent (and processed in parallel) or organized hierarchically (and processed along a neural hierarchy) (Friston and Buzsáki, 2016; Mirza et al., 2016).

In relation with the relevance of our model for how categorization is realized in the brain, we stress that our level of analysis is computational, according to the influential Marr’s taxonomy. Though our proposal is relevant for both algorithmic and implementation (i.e., neural) levels, we stress that there is no necessary one-to-one mapping between different levels of analysis. In other words, algorithmic or computational schemes need to be consistent with a normative proposal, but more than one scheme can fit with that proposal. Indeed, we have covertly appealed to very diverse mechanisms (experience-dependent plasticity during neurodevelopment and natural selection during evolution) in mediating Bayesian model selection – and the minimization of complexity.

A remarkable neuropsychological observation is that brain damage sometimes induces category-specific impairments (Warrington and McCarthy, 1983, 1987; Warrington and Shallice, 1984). For example, some patients exhibit categorization deficits specifically for living but not for non-living things, or *vice versa*. Sensory-functional theory (Warrington and McCarthy, 1983, 1987; Warrington and Shallice, 1984) explains these findings by distinguishing between visual/perceptual and associative/functional subsystems in the brain, with the former involved in categorizing living things and the latter in categorizing non-living things. This view stands in contrast to the domain-specific hypothesis (Caramazza and Shelton, 1998; Caramazza and Mahon, 2003) which explains category-specific deficits in terms of pre-wired neural circuits for processing innate categories such as “animals,” “fruits/vegetables,” “conspecifics,” and “tools.” Overall, the key difference between sensory-functional and domain-specific theory relies on the emphasis on ontogenetic and phylogenetic processes, respectively. While this debate is largely a matter of empirical research, the theory we propose here is potentially consistent with both views. In fact, our proposal is that learning is Bayesian (with all the ensuing implications presented here), but it does not preclude a substantial contribution to learning from evolution (i.e., optimization of the generative model through natural (Bayesian model) selection, as implied by domain-specific theory) in addition to a role played by experience (i.e., optimization of the generative model through experience dependent learning, as implied by sensory-functional theory). In other words, Bayesian category learning (at least at some level) may proceed at both an *evolutionary* and *somatic* timescales, a core premise of co-evolutionary theory (Kauffman and Johnsen, 1991; Rosenman and Saunders, 2003; Traulsen et al., 2006).

Although evolution may play an important role (for example, providing the prior knowledge before experience is accumulated), our theory stresses that categories are largely experience-dependent. Before a generative model can be used effectively to perform inference, its structure and parameters need to be learnt from experience. Though how this occurs is still poorly understood (Acuna and Schrater, 2009; Dolan and Dayan, 2013; Pezzulo et al., 2013, 2015, 2016; FitzGerald et al., 2014; Friston et al., 2016) and falls beyond our present remit; we conceive of learning as a model comparison or selection process that eventually favors a generative model with the characteristics of simplicity and accuracy; i.e., self evidencing (Hohwy, 2016). Model comparison based upon model evidence (or free energy) implies an optimal accuracy-complexity trade-off. While the burden associated with complexity can be intuitively connected with factors like metabolic and time costs, a more fundamental problem is overfitting, which is a pure statistical concept. In other words – in Bayesian statistics – complexity is penalized simply because a model with too many parameters explains new data poorly. The approximate Bayesian inference, self-evidencing premise we adopt is that complexity minimizing processes are implemented in the brain. For example, this perspective has been pursued to explain the synaptic homoeostasis seen during sleep (Tononi and Cirelli, 2006). See Hobson and Friston (2012) for a full discussion of synaptic regression and complexity minimization in this context.

In summary, we propose a theory of categorization based on the principle that the brain performs Bayesian inference to achieve goals. This theory integrates motivational and perceptual aspects within a unifying framework, and, from the Bayesian principle of trading off model accuracy and complexity, derives important aspects of categorization; such as the inclusion of latent variables, a hierarchical organization, and the explicit representation of context and action. The formulation may help in conceptualizing the computational functions of categorization and their link with brain structure and function.

## Author Contributions

All authors listed, have made substantial, direct and intellectual contribution to the work, and approved it for publication.

## Conflict of Interest Statement

The authors declare that the research was conducted in the absence of any commercial or financial relationships that could be construed as a potential conflict of interest.

## References

[B1] Acuna D. Schrater P. R. (2009). “Structure learning in human sequential decision-making,” in *Proceedings of the 2008 Conference: Advances in Neural Information Processing Systems 21* Vancouver, BC 1–8.

[B2] Anderson J. R. (1990). *The Adaptive Character of Thought.* Hillsdale, NJ: Erlbaum.

[B3] Anthony M. Bartlett P. L. (2009). *Neural Network Learning: Theoretical Foundations.* Cambridge: Cambridge university press.

[B4] Ashby F. G. Gott R. E. (1988). Decision rules in the perception and categorization of multidimensional stimuli. *J. Exp. Psychol.* 14 33–53. 10.1037/0278-7393.14.1.33 2963894

[B5] Ashby F. G. Townsend J. T. (1986). Varieties of perceptual independence. *Psychol. Rev.* 93 154–179. 10.1037/0033-295X.93.2.154 3714926

[B6] Barsalou L. W. (1983). Ad hoc categories. *Mem. Cogn.* 11 211–227. 10.3758/BF03196968 6621337

[B7] Barsalou L. W. (2008). Grounded cognition. *Annu. Rev. Psychol.* 59 617–645. 10.1146/annurev.psych.59.103006.093639 17705682

[B8] Barsalou L. W. Simmons W. K. Barbey A. K. Wilson C. D. (2003). Grounding conceptual knowledge in modality-specific systems. *Trends Cogn. Sci.* 7 84–91. 10.1016/S1364-6613(02)00029-3 12584027

[B9] Barto A. G. Mahadevan S. (2003). Recent advances in hierarchical reinforcement learning. *Discrete Event Dyn. Syst.* 13 41–77. 10.1023/A:1022140919877

[B10] Bishop C. M. (2006). *Pattern Recognition and Machine Learning.* Berlin: Springer.

[B11] Botvinick M. Toussaint M. (2012). Planning as inference. *Trends Cogn. Sci.* 16 485–488. 10.1016/j.tics.2012.08.006 22940577

[B12] Botvinick M. M. Niv Y. Barto A. C. (2009). Hierarchically organized behavior and its neural foundations: a reinforcement learning perspective. *Cognition* 113 262–280. 10.1016/j.cognition.2008.08.011 18926527 PMC2783353

[B13] Bouton M. E. (1993). Context, time, and memory retrieval in the interference paradigms of Pavlovian learning. *Psychol. Bull.* 114 80–99. 10.1037/0033-2909.114.1.80 8346330

[B14] Caramazza A. Mahon B. Z. (2003). The organization of conceptual knowledge: the evidence from category-specific semantic deficits. *Trends Cogn. Sci.* 7 354–361. 10.1016/S1364-6613(03)00159-1 12907231

[B15] Caramazza A. Shelton J. R. (1998). Domain-specific knowledge systems in the brain: the animate-inanimate distinction. *J. Cogn. Neurosci.* 10 1–34. 10.1162/089892998563752 9526080

[B16] Chater N. Tenenbaum J. B. Yuille A. (2006). Probabilistic models of cognition: conceptual foundations. *Trends Cogn. Sci.* 10 287–291. 10.1016/j.tics.2006.05.007 16807064

[B17] Clark A. (2013). Whatever next? Predictive brains, situated agents, and the future of cognitive science. *Behav. Brain Sci.* 36 181–204. 10.1017/S0140525X12000477 23663408

[B18] Collins A. G. Frank M. J. (2013). Cognitive control over learning: creating, clustering, and generalizing task-set structure. *Psychol. Rev.* 120 190–229. 10.1037/a0030852 23356780 PMC3974273

[B19] Collins A. M. Quillian M. R. (1969). Retrieval time from semantic memory. *J. Verbal Learning Verbal Behav.* 8 240–247. 10.1016/S0022-5371(69)80069-1

[B20] Courville A. C. Daw N. D. Touretzky D. S. (2006). Bayesian theories of conditioning in a changing world. *Trends Cogn. Sci.* 10 294–300. 10.1016/j.tics.2006.05.004 16793323

[B21] Dayan P. Hinton G. E. Neal R. M. Zemel R. S. (1995). The helmholtz machine. *Neural Comput.* 7 889–904. 10.1162/neco.1995.7.5.889 7584891

[B22] Dickinson A. Balleine B. (1994). Motivational control of goal-directed action. *Anim. Learn. Behav.* 22 1–18. 10.3758/BF03199951

[B23] Dolan R. J. Dayan P. (2013). Goals and habits in the brain. *Neuron* 80 312–325. 10.1016/j.neuron.2013.09.007 24139036 PMC3807793

[B24] Estes W. K. (1994). *Classification and Cognition.* New York, NY: Oxford University Press 10.1093/acprof:oso/9780195073355.001.0001

[B25] FitzGerald T. H. Dolan R. J. Friston K. J. (2014). Model averaging, optimal inference, and habit formation. *Front. Hum. Neurosci.* 8:457 10.3389/fnhum.2014.00457 PMC4071291 25018724

[B26] Friston K. (2005). A theory of cortical responses. *Philos. Trans. R. Soc. B.* 360 815–836. 10.1098/rstb.2005.1622 PMC1569488 15937014

[B27] Friston K. (2010). The free-energy principle: a unified brain theory? *Nat. Rev. Neurosci.* 11 127–138. 10.1038/nrn2787 20068583

[B28] Friston K. (2012). The history of the future of the Bayesian brain. *Neuroimage* 62 1230–1233. 10.1016/j.neuroimage.2011.10.004 22023743 PMC3480649

[B29] Friston K. Buzsáki G. (2016). The functional anatomy of time: what and when in the brain. *Trends Cogn. Sci.* 20 500–511. 10.1016/j.tics.2016.05.001 27261057

[B30] Friston K. FitzGerald T. Rigoli F. Schwartenbeck P. Pezzulo G. (2016). Active inference and learning. *Neurosci. Biobehav. Rev.* 68 862–879. 10.1016/j.neubiorev.2016.06.022 27375276 PMC5167251

[B31] Friston K. Rigoli F. Ognibene D. Mathys C. Fitzgerald T. Pezzulo G. (2015). Active inference and epistemic value. *Cogn. Neurosci.* 6 187–214. 10.1080/17588928.2015.1020053 25689102

[B32] Friston K. Schwartenbeck P. FitzGerald T. Moutoussis M. Behrens T. Dolan R. J. (2013). The anatomy of choice: active inference and agency. *Front. Hum. Neurosci.* 7:598 10.3389/fnhum.2013.00598 PMC3782702 24093015

[B33] Gershman S. J. Niv Y. (2010). Learning latent structure: carving nature at its joints. *Curr. Opin. Neurobiol.* 20 251–256. 10.1016/j.conb.2010.02.008 20227271 PMC2862793

[B34] Griffiths T. L. Canini K. R. Sanborn A. N. Navarro D. J. (2007). “Unifying rational models of categorization via the hierarchical Dirichlet process,” in *Proceedings of the 29th Annual Cognitive Science Society* Nashville, TN 323–328.

[B35] Hinton G. E. Osindero S. Teh Y. W. (2006). A fast learning algorithm for deep belief nets. *Neural Comput.* 18 1527–1554. 10.1162/neco.2006.18.7.1527 16764513

[B36] Hobson J. A. Friston K. J. (2012). Waking and dreaming consciousness: neurobiological and functional considerations. *Prog. Neurobiol.* 98 82–98. 10.1016/j.pneurobio.2012.05.003 22609044 PMC3389346

[B37] Hoeting J. A. Madigan D. Raftery A. E. Volinsky C. T. (1999). Bayesian model averaging: a tutorial. *Stat. Sci* 14 382–401.

[B38] Hohwy J. (2016). The self-evidencing brain. *Noûs* 50 259–285. 10.1111/nous.12062

[B39] Homa D. Sterling S. Trepel L. (1981). Limitations of exemplar-based generalization and the abstraction of categorical information. *J. Exp. Psychol.* 7 418–439. 10.1037/0278-7393.7.6.418

[B40] Kauffman S. A. Johnsen S. (1991). Coevolution to the edge of chaos: coupled fitness landscapes, poised states, and coevolutionary avalanches. *J. Theor. Biol.* 149 467–505. 10.1016/S0022-5193(05)80094-3 2062105

[B41] Knill D. C. Pouget A. (2004). The Bayesian brain: the role of uncertainty in neural coding and computation. *Trends Neurosci.* 27 712–719. 10.1016/j.tins.2004.10.007 15541511

[B42] Lamberts K. (2000). Information-accumulation theory of speeded categorization. *Psychol. Rev.* 107 227–260. 10.1037/0033-295X.107.2.227 10789196

[B43] Maddox W. T. Ashby F. G. (1993). Comparing decision bound and exemplar models of categorization. *Percept. Psychophys.* 53 49–70. 10.3758/BF03211715 8433906

[B44] McClelland J. L. Rogers T. T. (2003). The parallel distributed processing approach to semantic cognition. *Nat. Rev. Neurosci.* 4 310–322. 10.1038/nrn1076 12671647

[B45] Miller G. A. Galanter E. Pribram K. H. (1960). *Plans and the Structure of Behavior.* New York, NY: Henry Holt and Company 10.1037/10039-000

[B46] Mirza M. B. Adams R. A. Mathys C. D. Friston K. J. (2016). Scene construction, visual foraging and active inference. *Front. Comput. Neurosci.* 10:56 10.3389/fncom.2016.00056 PMC4906014 27378899

[B47] Nosofsky R. M. (1986). Attention, similarity, and the identification–categorization relationship. *J. Exp. Psychol.* 115 39–61. 10.1037/0096-3445.115.1.39 2937873

[B48] Oaksford M. Chater N. (2007). *Bayesian Rationality: The Probabilistic Approach to Human Reasoning.* Oxford: Oxford University Press 10.1093/acprof:oso/9780198524496.001.0001 19210833

[B49] Pezzulo G. Calvi G. (2011). Computational explorations of perceptual symbol systems theory. *New Ideas Psychol.* 29 275–297. 10.1016/j.newideapsych.2009.07.004

[B50] Pezzulo G. Cartoni E. Rigoli F. Pio-Lopez L. Friston K. (2016). Active inference, epistemic value, and vicarious trial and error. *Learn. Mem.* 23 322–338. 10.1101/lm.041780.116 27317193 PMC4918783

[B51] Pezzulo G. Rigoli F. (2011). The value of foresight: how prospection affects decision-making. *Front. Neurosci.* 5:79 10.3389/fnins.2011.00079 PMC3129535 21747755

[B52] Pezzulo G. Rigoli F. Chersi F. (2013). The mixed instrumental controller: using value of information to combine habitual choice and mental simulation. *Front. Psychol.* 4:92 10.3389/fpsyg.2013.00092 PMC3586710 23459512

[B53] Pezzulo G. Rigoli F. Friston K. (2015). Active Inference, homeostatic regulation and adaptive behavioural control. *Prog. Neurobiol.* 134 17–35. 10.1016/j.pneurobio.2015.09.001 26365173 PMC4779150

[B54] Rigoli F. Friston K. J. Dolan R. J. (2016a). Neural processes mediating contextual influences on human choice behaviour. *Nat. Commun.* 7:12416 10.1038/ncomms12416 PMC4992127 27535770

[B55] Rigoli F. Friston K. J. Martinelli C. Selaković M. Shergill S. S. Dolan R. J. (2016b). A Bayesian model of context-sensitive value attribution. *eLife* 5:e16127 10.7554/eLife.16127 PMC4958375 27328323

[B56] Rigoli F. Rutledge R. B. Dayan P. Dolan R. J. (2016c). The influence of contextual reward statistics on risk preference. *NeuroImage* 128 74–84. 10.1016/j.neuroimage.2015.12.016 26707890 PMC4767216

[B57] Roach N. W. McGraw P. V. Whitaker D. J. Heron J. (2017). Generalization of prior information for rapid Bayesian time estimation. *Proc. Natl. Acad. Sci. U.S.A.* 114 412–417. 10.1073/pnas.1610706114 28007982 PMC5240697

[B58] Rosch E. Mervis C. B. Gray W. D. Johnson D. M. Boyes-Braem P. (1976). Basic objects in natural categories. *Cognit. Psychol.* 8 382–439. 10.1016/0010-0285(76)90013-X

[B59] Rosch E. H. (1973). Natural categories. *Cognit. Psychol.* 4 328–350. 10.1016/0010-0285(73)90017-0

[B60] Rosch E. H. (1975). Cognitive reference points. *Cognit. Psychol.* 7 532–547. 10.1016/0010-0285(75)90021-3

[B61] Rosenblueth A. Wiener N. Bigelow J. (1943). Behavior, purpose and teleology. *Philos. Sci.* 10 18–24. 10.1086/286788

[B62] Rosenman M. Saunders R. (2003). Self-regulatory hierarchical coevolution. *AIEDAM* 17 273–285. 10.1017/S089006040317401X

[B63] Shenhav A. Botvinick M. M. Cohen J. D. (2013). The expected value of control: an integrative theory of anterior cingulate cortex function. *Neuron* 79 217–240. 10.1016/j.neuron.2013.07.007 23889930 PMC3767969

[B64] Sjöberg J. Zhang Q. Ljung L. Benveniste A. Delyon B. Glorennec P. Y. (1995). Nonlinear black-box modeling in system identification: a unified overview. *Automatica* 31 1691–1724. 10.1016/0005-1098(95)00120-8

[B65] Smith J. D. Minda J. P. (1998). Prototypes in the mist: the early epochs of category learning. *J. Exp. Psychol.* 24 1411–1436. 10.1037/0278-7393.24.6.1411

[B66] Solway A. Botvinick M. M. (2012). Goal-directed decision making as probabilistic inference: a computational framework and potential neural correlates. *Psychol. Rev.* 119 120–154. 10.1037/a0026435 22229491 PMC3767755

[B67] Squire L. R. (1986). Mechanisms of memory. *Science* 232 1612–1619. 10.1126/science.3086978 3086978

[B68] Stoianov I. Genovesio A. Pezzulo G. (2015). Prefrontal goal codes emerge as latent states in probabilistic value learning. *J. Cogn. Neurosci.* 28 140–157. 10.1162/jocn_a_00886 26439267

[B69] Tononi G. Cirelli C. (2006). Sleep function and synaptic homeostasis. *Sleep Med. Rev.* 10 49–62. 10.1016/j.smrv.2005.05.002 16376591

[B70] Traulsen A. Claussen J. C. Hauert C. (2006). Coevolutionary dynamics in large, but finite populations. *Phys. Rev. E* 74:11901 10.1103/PhysRevE.74.011901 16907121

[B71] Tulving E. (2002). Episodic memory: from mind to brain. *Annu. Rev. Psychol.* 53 1–25. 10.1146/annurev.psych.53.100901.135114 11752477

[B72] Tulving E. Thomson D. M. (1971). Retrieval processes in recognition memory: effects of associative context. *J. Exp. Psychol.* 87 116–124. 10.1037/h0030186

[B73] Warrington E. K. (1975). The selective impairment of semantic memory. *Q. J. Exp. Psychol.* 27 635–657. 10.1080/14640747508400525 1197619

[B74] Warrington E. K. McCarthy R. (1983). Category specific access dysphasia. *Brain* 106 859–878. 10.1093/brain/106.4.859 6652466

[B75] Warrington E. K. McCarthy R. A. (1987). Categories of knowledge. *Brain* 110 1273–1296. 10.1093/brain/110.5.1273 3676701

[B76] Warrington E. K. Shallice T. (1984). Category specific semantic impairments. *Brain* 107 829–853. 10.1093/brain/107.3.829 6206910

